# Human DOPA Decarboxylase in Monoamine Metabolism, Pharmacology, and Synucleinopathies

**DOI:** 10.1021/acsbiomedchemau.6c00141

**Published:** 2026-08-26

**Authors:** Davide Chiasserini, Giovanni Bellomo, Pier Luigi Orvietani, Mirco Dindo

**Affiliations:** † Department of Medicine and Surgery, 9309 University of Perugia, Piazza Lucio Severi 1, 06132 Perugia, Italy; ‡ Department of Life Science, Health, and Health Professions, Link Campus University, Via del Casale di San Pio V 44, 00165 Rome, Italy

**Keywords:** DOPA decarboxylase, α-synuclein, carbidopa, synucleinopathies, Parkinson’s disease, pyridoxal 5′-phosphate, fluid biomarker, gut microbiota

## Abstract

Human DOPA decarboxylase is the pyridoxal 5′-phosphate-dependent enzyme that produces dopamine and serotonin. For most of its history it has been treated as a constitutive downstream step rather than a point of regulation, and it has received correspondingly little mechanistic attention. The human enzyme is broadly expressed across central monoaminergic neurons and peripheral neuroendocrine tissues, where it operates in different chemical environments and under different pharmacological constraints. Structurally, it interconverts between an open, cofactor-free apo-form and a closed, catalytically competent holo-form, with conformational dynamics that extend well beyond the active site. Its activity is further tuned by cofactor availability, by phosphorylation, and by protein partners, of which the most notable is α-synuclein Its effect on the enzyme is documented in cellular systems but still unresolved at the molecular level. Pharmacologically, carbidopa is its principal peripheral inhibitor, but its mechanism remains incompletely characterized, and chronic therapy raises rather than lowers circulating AADC levels. In parallel, AADC measured in cerebrospinal fluid has emerged as one of the most discriminating fluid biomarkers for Parkinson’s disease and dementia with Lewy bodies, with changes detectable years before motor onset. These different layers, structural, regulatory, pharmacological, and diagnostic, converge on a single enzyme whose biology has long been treated as settled, and which is now being reevaluated as a fundamental node in the molecular landscape of synucleinopathies.

## Graphical abstract

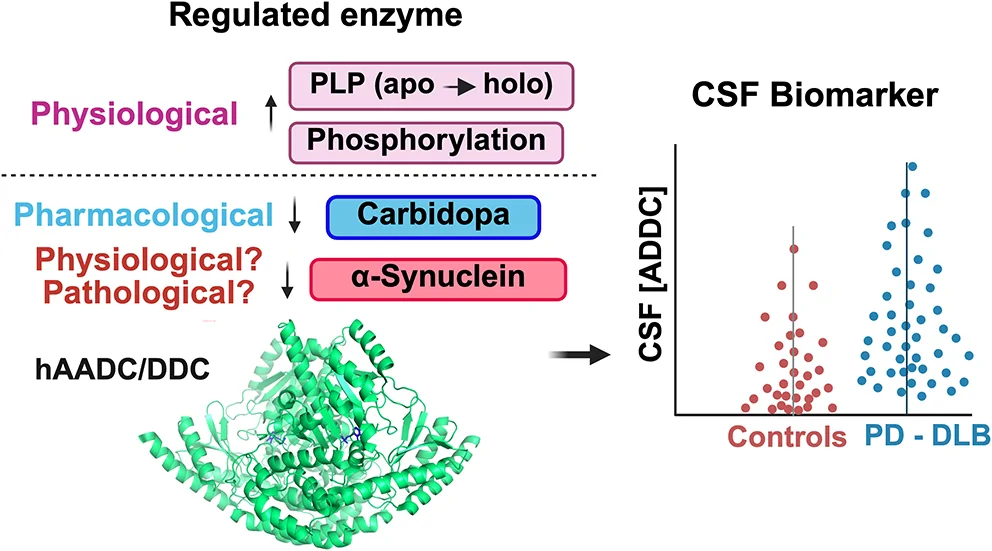

## Introduction

Human DOPA decarboxylase (also known as DDC or aromatic L-amino acid decarboxylase, AADC, EC 4.1.1.28) is the pyridoxal 5′-phosphate (PLP)-dependent enzyme that converts L-3,4-dihydroxyphenylalanine (L-DOPA) into dopamine and 5-hydroxytryptophan (5-HTP) into serotonin^1,2^ (Figure 1). Beyond its role as the target of carbidopa in Parkinson’s disease therapy, loss-of-function variants of the enzyme cause AADC deficiency, a rare autosomal recessive disorder of catecholamine and serotonin biosynthesis.^3–6^ For most of its history AADC has received only a fraction of the mechanistic attention reserved for tyrosine hydroxylase, the rate-limiting enzyme step upstream in the metabolic pathway leading to catecholamine biosynthesis. However, recent work has steadily undermined that picture. Human AADC structure has been resolved in apo, holo, and carbidopa-bound states, revealing an asymmetric dimer that undergoes large-scale conformational rearrangements upon cofactor binding^7–9^ (Figure 1). In parallel, proteomic analyses of cerebrospinal fluid have identified AADC as one of the most discriminating biomarkers for Parkinson’s disease (PD) and dementia with Lewy bodies (DLB), separating these conditions from healthy controls and from Alzheimer’s disease with excellent diagnostic performance; these changes are detectable in prodromal individuals with positive α-synuclein (α-syn) seeding assays, years before the onset of motor symptoms.^10–12^

**Figure 1:**
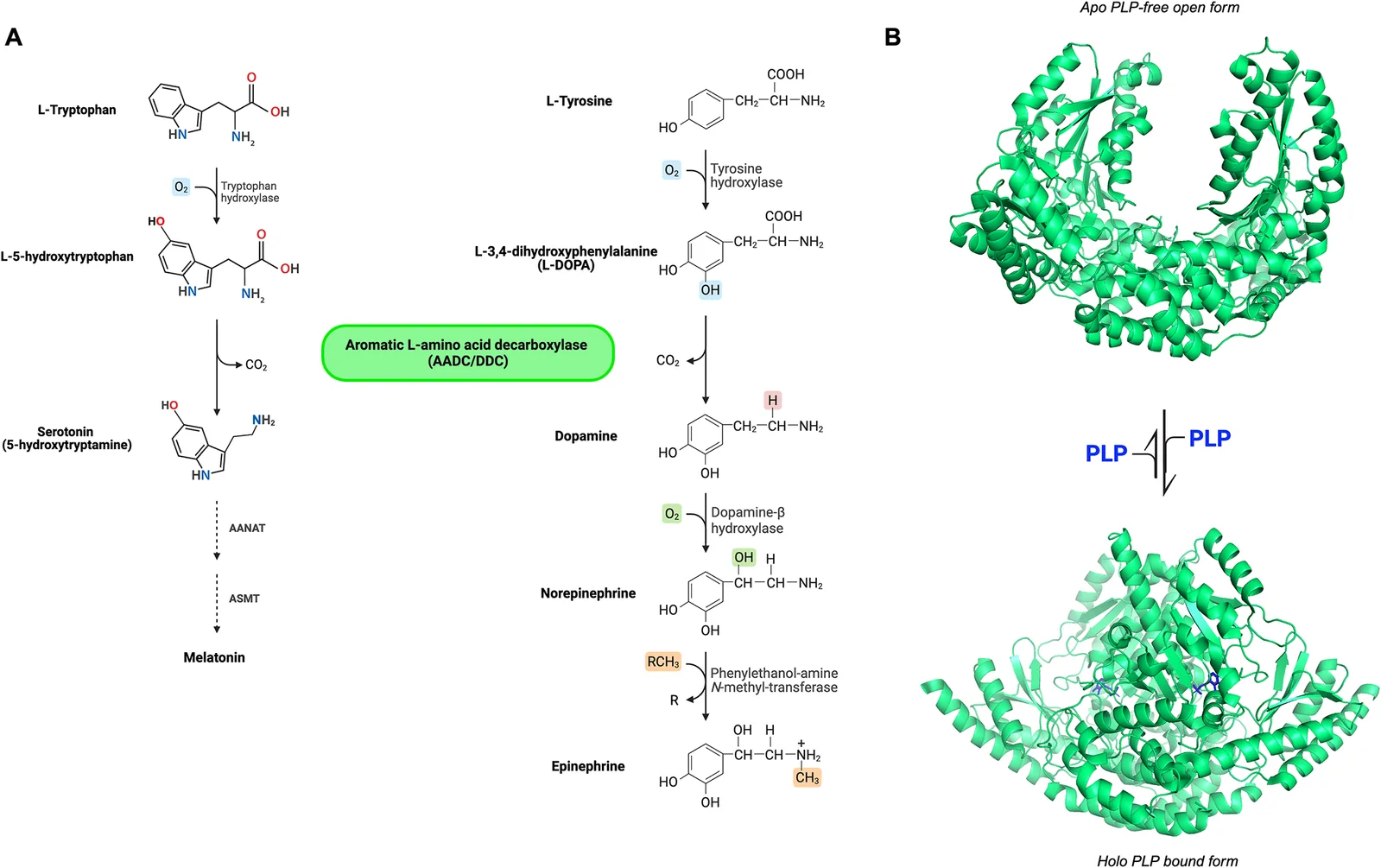
Aromatic L-amino acid decarboxylase (AADC) as the shared decarboxylation node of monoamine biosynthesis, and its cofactor-dependent conformational equilibrium. (a) AADC catalyzes the pyridoxal 5′-phosphate (PLP)-dependent decarboxylation that converts L-5-hydroxytryptophan to serotonin and L-3,4-dihydroxyphenylalanine (L-DOPA) to dopamine, removing the carboxyl group in both reactions. The aromatic amino acid precursors are generated upstream by tryptophan hydroxylase and tyrosine hydroxylase; downstream, dopamine β-hydroxylase and phenylethanolamine *N*-methyltransferase convert dopamine to norepinephrine and norepinephrine to epinephrine, respectively. AADC thus sits at the convergence point of the serotonergic and catecholaminergic pathways.^13^ (b) Cofactor-driven conformational change of human AADC. In the absence of PLP the enzyme adopts an open, catalytically inactive apo-conformation (top; PDB 3RBL).^7^ PLP binding drives closure to the compact, catalytically competent holo-form (bottom; PDB 8OR9),^8^ with the cofactor (blue) bound at each of the two active sites located at the dimer interface. Panel (a) has been redrawn with the correct indole connectivity.

A separate line of work has shown that the bacterial AADC homologues of the gut microbiota share substrate specificity with the human enzyme but differ sharply in their pharmacological vulnerability, a point developed in the section on the gut. These lines of work describe the same enzyme from complementary angles, and together they depart from the picture of an inert downstream catalyst. Several factors modulate AADC availability, post-translational modifications, and protein–protein interactions (such as with α-syn). Additionally, the tissue context could play a key role. Central and enteric neurons, the adrenal medulla, enterochromaffin cells, and renal tubules all express the same gene, yet operate under distinct chemical and pharmacological constraints/environments. What emerges is an enzyme whose activity is governed by the molecular form it takes and the tissue where it acts, and it is this regulated state, shaped by dopaminergic therapy, that the fluid biomarker signal in synucleinopathies ultimately reflects.

## Structural and Catalytic Framework of Human AADC

Human AADC (hAADC) is a homodimeric, PLP-dependent enzyme belonging to the type II family of PLP-dependent decarboxylases (Fold Type I, group II), which also includes glutamate decarboxylase, histidine decarboxylase, and bacterial aromatic amino acid decarboxylases. The active site is generated at the interface between the two subunits and contains residues from both monomers.^1,14^ In the holoenzyme, the PLP cofactor is anchored at the active site through a Schiff base (internal aldimine) with the ε-amino group of Lys303.^15^ Spectroscopic analyses of hAADC show absorbance maxima at 420 and 335 nm and fluorescence emission at 384 and 504 nm; titrations of the bound cofactor identify a single spectroscopic p*K*_*a*_ of 7.2 governing the equilibrium between the low- and high-pH forms of the internal aldimine.^16^ Stopped-flow analysis of the reaction with L-DOPA allows to identify spectral intermediates of the external aldimine, whose pH-dependent interconversion (p*K* 6.4) matches the value seen in the steady-state kinetic parameters, linking the spectroscopic transition to the rate-determining catalytic step.^16^ Beyond its two physiological substrates, L-DOPA and 5-HTP, hAADC also decarboxylates other aromatic L-amino acids, and catalyzes alternative side reactions on serotonin-including half-transamination and oxidative deamination-at distinct rates under aerobic and anaerobic conditions.^17^ These secondary activities point to an intrinsic chemical plasticity of the catalytic pocket that extends beyond unidirectional decarboxylation.

### Dimer Architecture and the Open/Closed Conformational Equilibrium

The conformational plasticity inferred from kinetic studies on AADC has a structural counterpart, revealed through four stages of crystallographic work. The first structures were obtained on pig kidney AADC. Burkhard and colleagues solved the ligand-free enzyme and its carbidopa complex (PDB 1JS3),^18^ showing that the inhibitor forms a hydrazone linkage with the PLP cofactor and establishing that the enzyme adopts the canonical architecture of the type II decarboxylase fold. These data remained the only available working model for the human enzyme for over a decade.^18^ Next, Giardina G. and colleagues solved the structure of hAADC in an unexpected, fully open conformation in which the two subunits move 20 Å apart, fully exposing the active sites; by tuning PLP concentration in the crystals they also captured intermediate states of the open/closed transition^7^ (Figure 1). These data established PLP binding as the structural switch that reorganizes the dimer interface, linking cofactor availability directly to enzyme stability, based on the increased susceptibility to degradation of the apo-form.^7^ In 2023, Bisello and colleagues^8^ reported the first holo-hAADC structures, capturing both the internal aldimine (PDB 8OR9) and the external aldimine with L-DOPA methylester (PDB 8ORA). Combining crystallography with molecular dynamics, SAXS, and limited proteolysis, they showed that the obligate dimer is elongated and asymmetric, with catalytic-loop fluctuations coupled to flexibility at the N/C-domain edge. Tellingly, AADC-deficiency variants that map far from the active site, yet compromise loop flexibility, were found to impair catalysis, confirming that the integrity of this peripheral region is essential for activity.^8^ Most recently, Baine and colleagues re-examined the human enzyme bound to carbidopa at high resolution (PDB 9GNS), assigning the inhibitor adduct to the catalytically relevant ketoenamine tautomer of the PLP-hydrazone, with parallel structures across group II decarboxylases supporting the generality of this model.^9^ Three features demonstrate that hAADC exists in a defined apo–holo equilibrium governed by large-scale subdomain motions, with cofactor availability modulating stability; the dimer is asymmetric and dynamically coupled and its catalytic-loop flexibility tuned by elements far from the active site.

## Tissue Distribution and Isoforms

Although AADC is most strongly associated with central catecholaminergic and serotonergic neurons, its expression is considerably broader. In the central nervous system, the enzyme is expressed in dopaminergic neurons of the substantia nigra pars compacta and ventral tegmental area, as well as in serotonergic neurons of the dorsal raphe nucleus and in noradrenergic neurons of the locus coeruleus.^19^ In the periphery, AADC is concentrated in neuroendocrine tissues. Adrenal medullary chromaffin cells synthesize the systemic catecholamine pool, and human AADC was historically purified from pheochromocytoma tissue.^20^ In the gastrointestinal tract, enterochromaffin cells produce the bulk of peripheral serotonin and constitute the main extra-neuronal source of AADC activity;^21^ the enzyme is also present in enteric neurons, where it supports local dopamine and serotonin synthesis.^22^ Beyond neuroendocrine cells, AADC is detected in human kidney, liver, pancreas, placenta, leukocytes and T-lymphocytes,^23^ with the highest non-neuronal expression found in the renal proximal tubules.^24^ The human AADC gene generates distinct neuronal and non-neuronal mRNA transcripts through alternative splicing of the 5′-untranslated region, although the protein product is identical.^25^ A C-terminally truncated isoform (Alt-AADC) of approximately 37 kDa has also been described, with preferential expression in peripheral tissues; its catalytic properties and physiological role remain uncharacterized.^26^

## Modulators of AADC Activity

### Cofactor Availability and Post-Translational Control

The catalytic output of AADC is shaped by three main inputs represented by cofactor availability, post-translational modification, and the binding of cellular partners (Figure 2). These effects are most apparent in the striatum, where AADC activity responds to pharmacological manipulation and is altered in animal models of nigrostriatal denervation.^27^ hAADC exists in equilibrium between an open, PLP-free apo-form and a closed, catalytically competent holo-form.^7^ Free intracellular PLP is kept very low to limit toxicity, and the enzyme is thought to acquire its cofactor through transient contact with the B6-salvage machinery. In vitro, pyridoxine 5′-phosphate oxidase (PNPO) binds both forms of AADC and hands its bound PLP to the apoenzyme, partially reconstituting active holoenzyme.^28^ The availability of vitamin B6 thus governs how much of the cellular AADC pool is catalytically competent. Phosphorylation provides a second layer of control by activating the enzyme and is itself responsive to the surrounding neurotransmitter tone.^27,29^ A specific target on the human enzyme has recently been identified-PKA phosphorylates Ser193, a conserved active-site residue, increasing catalytic efficiency toward L-DOPA and 5-HTP largely through a decrease in Km.^30^ Phosphomimetic substitutions do not reproduce this effect, leaving the molecular basis of the activation open; in addition, phosphorylation of Ser193 in a cellular environment has not yet been demonstrated. Whether the modification regulates human AADC in vivo remains to be established, but the identification of a defined activating phosphosite turns this into a testable mechanism rather than an inference from other species.

**Figure 2:**
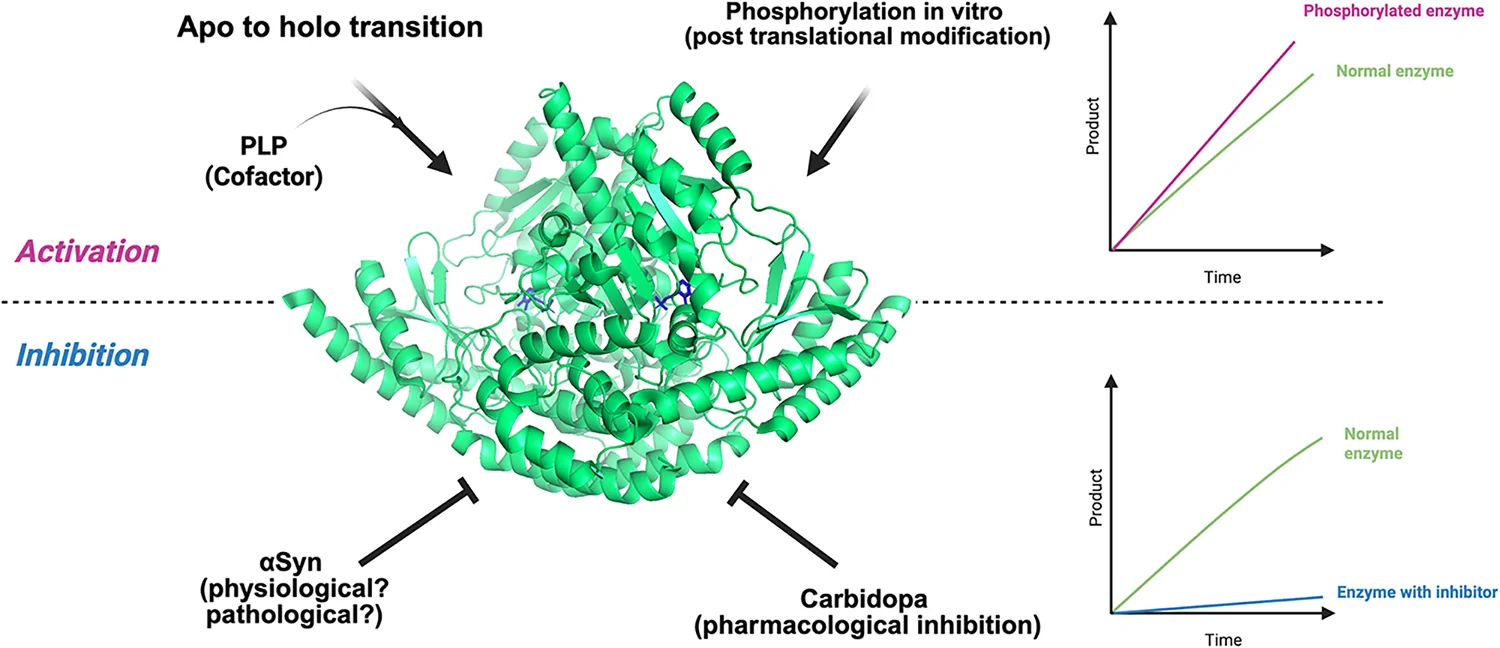
Human AADC as a finely modulated enzyme. The catalytically competent holo-enzyme (center; pyridoxal 5′-phosphate, PLP, shown in blue at the two active sites) is subject to several regulatory inputs, grouped here into those that increase activity (above the dashed line) and those that decrease it (below). Activation: binding of the cofactor PLP drives the apo-to-holo transition required for catalysis, and phosphorylation, a post-translational modification shown to act on the human enzyme in vitro, increases catalytic efficiency. Inhibition: α-syn lowers enzyme activity through a protein–protein interaction whose physiological versus pathological role remains to be defined, and the peripheral inhibitor carbidopa inhibits the enzyme. The schematic progress curves (product versus time) illustrate the expected kinetic consequences, where phosphorylation raises the enzyme performance relative to the unmodified enzyme (upper curves, magenta versus green), whereas an inhibitor strongly suppresses it (lower curves, blue versus green). Question marks denote interactions whose physiological relevance, or whose effect on the human enzyme in vivo, has not yet been established.

### α-Syn as Protein Partner

The role of AADC in dopamine production at the presynaptic terminal might be intertwined with the function of α-syn in neurons. Although the function of α-syn in the synapse can still be considered unresolved, several lines of evidence have shown that this protein might be involved in neurotransmitter vesicle trafficking.^31^ It seems reasonable to hypothesize that the two proteins may interact at the synapse, contributing to the regulation of dopaminergic tone.^32^ Indeed, AADC has been shown to functionally interact with α-syn (Figure 2). In MN9D and PC12 dopaminergic cells that overexpress wild-type or A53T α-syn, Tehranian et al. showed that α-syn does not change AADC protein levels but reduces both its activity and its phosphorylation on serine residues, with coimmunoprecipitation confirming an interaction in rat striatum.^33^ The effect was comparable for wild-type and A53T forms, and affected both L-DOPA and 5-HTP as substrates. The proposed mechanism, α-syn-mediated activation of protein phosphatase 2A (PP2A) and consequent dephosphorylation of AADC, parallels that described for tyrosine hydroxylase by the same group,^34^ and is supported by the observation that other substrates of PP2A such as Akt are not affected, indicating that dephosphorylation is selective for partners interacting with α-syn rather than reflecting general activation of phosphatase. The specific serine residue(s) modulated in cells were not identified, and whether the activating Ser193 phosphorylation site characterized in vitro on the human enzyme^30^ is the same residue dephosphorylated by α-syn-driven PP2A activity remains an open question.

This interaction is usually framed in the context of pathology, since α-syn is the principal component of Lewy bodies (LBs)^38^ and AADC colocalizes with phosphorylated α-syn in LBs.^10^ It is worth noting, however, that α-syn and AADC are coexpressed well beyond the degenerating dopaminergic neurons (Figure 3). Both are present in central catecholaminergic and serotonergic neurons; both are found in enteric neurons, which express α-syn essentially throughout the enteric nervous system;^39^ and both occur in gut epithelial enteroendocrine and enterochromaffin cells, which combine α-syn expression with neuron-like properties.^40^ This anatomical overlap suggests that the α-syn–AADC relationship may not be confined to disease, but could reflect a physiological interaction between two proteins that share the same cellular localization and functional environments. The current evidence for AADC-α-syn functional interaction is limited by mainly two factors. First, most studies have been carried out in overexpression systems with nonphysiological α-syn levels. Second, α-syn may be present in cellular systems under a variety of conformational states, disordered monomer, soluble oligomers, fibrils^41^ and whether these forms either engage hAADC differently or alter its catalytic properties, remains an open question. Given how extensively the two proteins are coexpressed in several tissues, clarifying this interaction at both physiological and pathological levels would help establish whether α-syn is a genuine regulator of AADC in vivo, and in which tissues and compartments.

**Figure 3:**
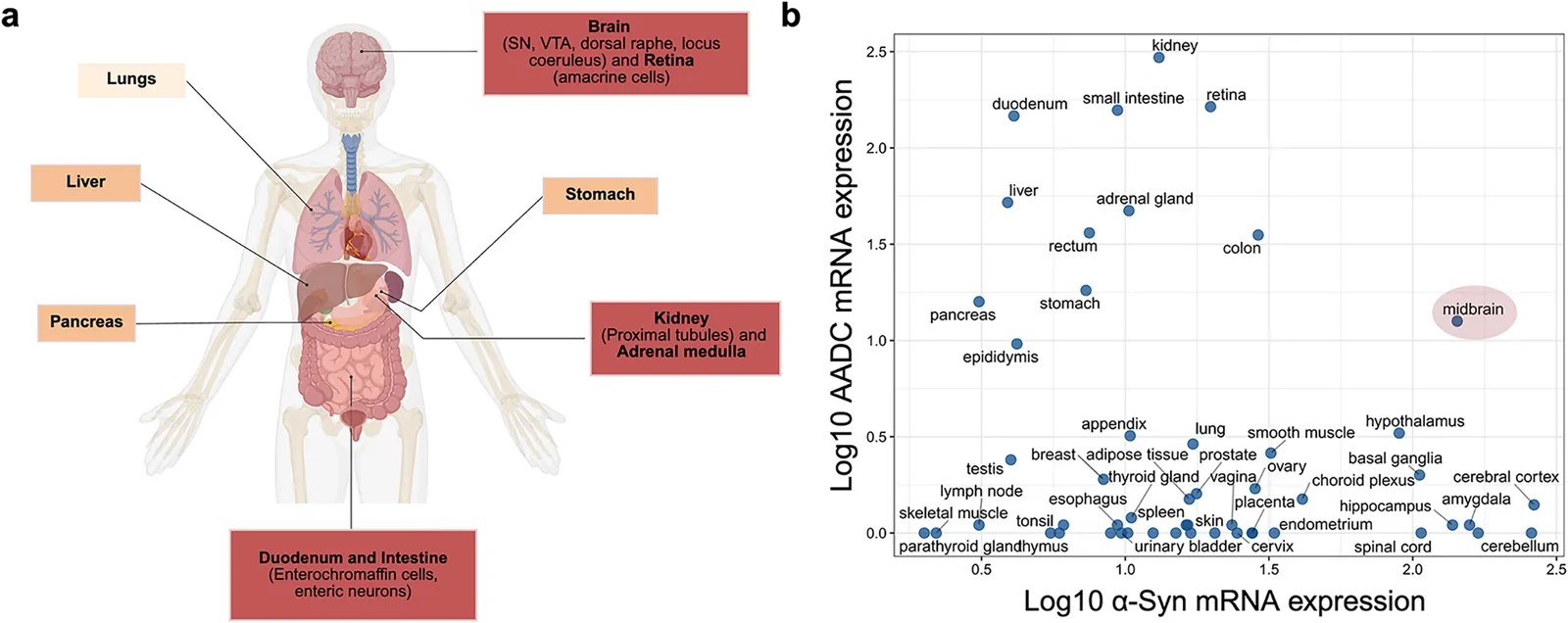
Tissue distribution of AADC in humans and its coexpression with α-syn. (a) Schematic representation of AADC expression across human tissues, color-coded by expression level. High expression (red boxes) is found in monoaminergic neurons of the central nervous system (substantia nigra pars compacta, ventral tegmental area, dorsal raphe nucleus, locus coeruleus);^19^ dopaminergic amacrine cells of the retina;^35^ enterochromaffin cells and enteric neurons of the duodenum and small intestine;^21,22^ adrenal medullary chromaffin cells, the source of systemic catecholamines from which the human enzyme was historically purified;^20^ and renal proximal tubular cells, the highest non-neuronal expression site.^24^ Moderate expression (amber boxes) is detected in liver, pancreas, stomach, and pulmonary neuroendocrine cells of the airway epithelium.^23,24,36,37^ (b) Normalized mRNA expression of AADC and α-syn across human tissues, retrieved from the Human Protein Atlas.^24^ AADC is predominantly expressed in gut tissues, kidney, and retina, whereas α-syn is highly expressed throughout the brain. Midbrain regions (raphe nuclei and substantia nigra) are the exception to this pattern, combining high α-syn expression with relatively high AADC expression, the anatomical context in which the AADC-α-syn interaction is most relevant to disease.

### The Unresolved Mechanism of Carbidopa as a Peripheral Inhibitor

Carbidopa (α-methyl-α-hydrazino-DOPA) which is coadministered with L-DOPA, blocks peripheral aromatic amino acid decarboxylation and increases central L-DOPA bioavailability. Since it does not cross the blood-brain barrier, its action is confined to peripheral tissues.^42^ Structurally, its α-hydrazino group condenses with the C4′-aldehyde of PLP to form a hydrazone adduct in the active site, first visualized in pig AADC^18^ (PDB 1JS3) and recently resolved at 1.93 Å for the human enzyme (PDB 9GNS), with the adduct refined as the catalytically relevant ketoenamine tautomer.^9^ The same study also followed the reaction of carbidopa with the human enzyme by UV/vis stopped-flow spectrophotometry, reporting a pseudo-second-order association rate constant of 3.7 × 10^2^ M^–1^ s^–1^ (pH 7.4, 25 °C), with inhibition developing over roughly 2−3 min. This value is roughly two to three orders of magnitude lower than the association rates reported in the same study for the analogous lysine inhibitors of a bacterial decarboxylase, and the authors suggest it may reflect steric hindrance from the α-methyl group.^9^

In the clinical and biochemical literature, carbidopa is classified under conflicting mechanisms. While some authors describe it as a classical competitive inhibitor,^43,44^ others characterize the drug as an irreversible inhibitor of pig kidney AADC.^45^ Furthermore, it is often defined more generally as a PLP-binding agent,^46,47^ a property that underlies its contribution to vitamin B6 depletion during chronic therapy. In fact, carbidopa lowers tissue pyridoxal phosphate levels by sequestering the cofactor as a hydrazone adduct.^48^

The association rate does not, however, determine the reversibility of the resulting adduct. The hydrazone linkage connects the inhibitor covalently to the cofactor, but a covalent adduct is not in itself a kinetic category. The return to free enzyme requires hydrolysis of the hydrazone, and the rate of that hydrolysis is not fixed by the rate at which the adduct forms. Hydrazones are in general reversible under physiological conditions, and hydrolysis releases PLP, which free carbidopa can in principle intercept in solution; sequestration of the cofactor in this way would limit reconstitution of the holo-enzyme, and could contribute to the apparent irreversibility observed in some settings independently of the affinity of the active-site adduct. The crystallographic work further shows that carbidopa engages the active site as the substrate would, with its α-carboxylate held in a Dunathan-like alignment 3.2 Å from the His192.^9^ A quantitative account of the inhibition therefore still requires an explicit determination of the dissociation rate, of the equilibrium inhibition constant, and of the drug-target residence time on recombinant human AADC. The analysis would ideally extend beyond dilute-buffer conditions. Indeed, it is well-known that macromolecular crowding modulates catalysis and protein–protein interactions^49–53^ and could modulate inhibitor binding. Liquid–liquid phase separated systems that reproduce these conditions^54,55^ now make this analysis feasible alongside classical bulk experiments. In addition, chronic L-DOPA/carbidopa therapy increases rather than depletes circulating AADC protein levels and enzyme activity,^46,56^ a response consistent with a dynamic inhibition equilibrium and compensatory upregulation rather than stable inactivation. Peripheral inhibition simultaneously impairs both dopamine and serotonin synthesis, with significant implications for the gut compartment (where host AADC, bacterial homologues and α-syn could interact).

## Peripheral AADC and Gut Microbiota

The gastrointestinal tract hosts two decarboxylase activities in parallel (Figure 4). The first is host AADC, which is highly expressed and second only to the kidney.^24^ The second is a bacterial AADC repertoire encoded by members of the gut microbiota.^57^ Both produce biogenic amines locally, but the peripheral decarboxylase inhibitors used in standard PD therapy act on them with very different efficiency.

**Figure 4:**
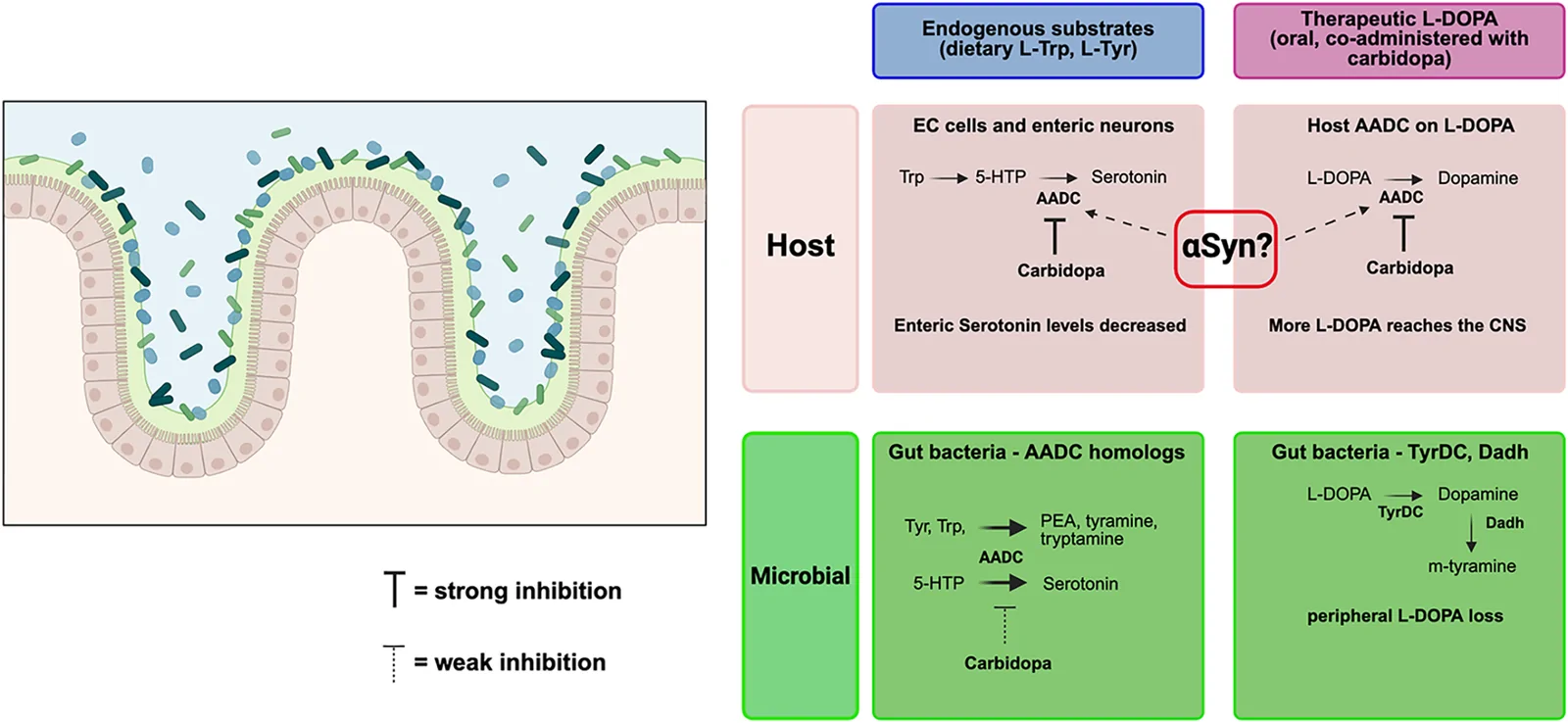
Host and microbial aromatic L-amino acid decarboxylases (AADC) act in parallel in the gut and are differentially engaged by carbidopa. The columns separate the two substrate sources. Endogenous dietary amino acids (l-Trp, l-Tyr; left) and orally administered therapeutic L-DOPA (coadministered with carbidopa) (right). The rows separate the host compartment (enterochromaffin cells and enteric neurons; upper) from the gut microbiota (lower). Host, endogenous substrates: enterochromaffin cells synthesize serotonin from tryptophan via 5-hydroxytryptophan (5-HTP), with AADC catalyzing the final decarboxylation step; carbidopa inhibits this step, so enteric serotonin synthesis is expected to fall. Host, therapeutic L-DOPA: carbidopa inhibits host AADC in the periphery, allowing more L-DOPA to escape peripheral decarboxylation and reach the central nervous system. Microbial, endogenous substrates: bacterial AADC homologues decarboxylate aromatic amino acids to aromatic amines (phenylethylamine, PEA; tyramine; tryptamine), and some commensals have been reported to produce serotonin from 5-HTP. In addition, these bacterial enzymes are only weakly and variably inhibited by carbidopa (dashed bar). Microbial, therapeutic L-DOPA: bacterial tyrosine decarboxylase (TyrDC) converts L-DOPA to dopamine, and a dehydroxylase (Dadh) further converts it to *m*-tyramine, diverting L-DOPA from absorption and contributing to peripheral L-DOPA loss. α-Syn, which is coexpressed with AADC in enteric neurons and enteroendocrine cells, has been shown to lower AADC activity in a dopaminergic cell model; whether the same modulation occurs at the enteric AADC, and whether it has physiological or pathological significance there, has not been characterized (dashed lines and question mark). Solid bars denote strong inhibition; dashed bars denote weak, species-dependent inhibition of bacterial enzymes; dashed arrows with a question mark denote a proposed but uncharacterized modulation.

This convergence has a direct consequence for serotonin that is easily overlooked. Most discussion of L-DOPA/carbidopa therapy is framed in terms of dopamine, yet AADC also catalyzes the final step of serotonin biosynthesis, the conversion of 5-hydroxytryptophan to serotonin. The periphery (not the brain) is where most of the body’s serotonin resides, with the large majority produced by enterochromaffin cells of the gut^21^ where serotonin regulates intestinal motility, secretion, and gut–brain signaling.^58^ Because carbidopa inhibits AADC without distinguishing between its two products, inhibition of the host enzyme would be expected to lower enteric serotonin synthesis alongside dopamine (Figure 4). This effect has received little attention despite the prominence of gastrointestinal dysfunction in PD. The role of the microbiota in this picture is real but indirect, and does not circumvent the host enzyme. Gut bacteria can raise host serotonin in two ways. They induce TPH1 expression in enterochromaffin cells^59^ and bacterial amines such as tryptamine evoke serotonin production from myenteric neurons.^60^ Both effects act upstream of the host pathway, whose final step still depends on host AADC. Carbidopa-mediated inhibition of host AADC therefore likely reduces enteric serotonin synthesis, although the magnitude of this effect in treated patients has not been characterized. The exception is the direct synthesis of serotonin by certain commensals. Indeed, some human gut bacteria have recently been shown to decarboxylate 5-hydroxytryptophan to serotonin and to modulate enteric serotonin and colon innervation, although this was demonstrated in gnotobiotic models, required bacterial communities rather than single strains, and remains to be established in humans.^61^ Production of serotonin has been reported in vitro for several other commensals as well,^60^ though direct evidence of a physiological contribution in humans is generally lacking. The net effect of chronic carbidopa on enteric serotonin is thus unresolved, but the underlying biochemistry points toward a reduction.

The bacterial decarboxylases are inhibited by carbidopa and benserazide far less effectively than the host enzyme, and in a species-dependent manner. Carbidopa is roughly 4 orders of magnitude more potent against human AADC than against a representative bacterial decarboxylase,^57,62^ suppressing amine production by some bacterial species while others remain largely unaffected. Conventional doses therefore inhibit host enteric AADC almost completely while leaving the microbial enzymes only partially affected. The enteric compartment has an additional significance in the context of synucleinopathy. The Braak staging hypothesis proposes that α-syn pathology may begin in the gut and ascend through the vagus nerve to the brainstem;^63^ although debated, it is supported by the observation that gastrointestinal symptoms such as constipation can precede motor symptoms by years, and that α-syn aggregates are found in the bowel of patients in the preclinical phase of the disease.^64^ The same enteric neurons and enteroendocrine cells that carry this early pathology also express AADC and α-syn,^40^ and are exposed (in patients treated for PD) to chronic carbidopa. Whether α-syn modulates enteric AADC in the way reported in dopaminergic cell models,^33^ and whether such a modulation has any physiological or pathological role in the gut, has not been established (Figure 4).

These observations describe a single peripheral system whose components have generally been studied separately. The bidirectional relationship between PD treatment and the gut microbiota is well documented. Bacteria metabolize levodopa, and the drugs in turn reshape microbial communities,^65^ and microbial signals have been linked to enteric α-syn aggregation in animal models.^66^ Limiting the bacterial degradation of levodopa alone or together with carbidopa, is itself an active area of work.^67^ What has not been examined as a system is the simultaneous pressure that chronic carbidopa places on host enteric AADC, on the bacterial decarboxylase repertoire, and on the aminergic environment in which α-syn aggregation may occur. Whether long-term carbidopa alters the microbial decarboxylase repertoire and luminal amine balance, whether host enteric AADC undergoes the compensatory rise seen in plasma, and whether the resulting environment influences enteric α-syn aggregation remain, to our knowledge, open questions.

## Aromatic L-Amino Acid Decarboxylase as Biomarker of Lewy Body Disorders

In recent years, AADC has emerged as a promising fluid biomarker for Lewy body disorders (LBDs), particularly Parkinson’s disease (PD) and dementia with Lewy bodies (DLB).^10,11^ Its functional role, its centrality in PD therapy, and its possible interaction with α-syn support a direct link between the biomarker signal and the underlying pathology. The principal studies investigating AADC in biofluids across neurodegenerative diseases are summarized in Table 1.

**Table 1:** Summary of Studies Investigating DOPA Decarboxylase (DDC) as a Biomarker in Lewy Body Disorders and Related Neurodegenerative Diseases^a^

Study	Year	Matrix	Assay	Comparison and key finding
Del Campo et al.	2023	CSF	PEA	DLB vs AD: best biomarker in a panel discriminating DLB from AD
Paslawski et al.	2023	CSF	PEA	PD/AP vs HC: increased in PD and AP, associated with dopaminergic treatment
Pereira et al.	2023	CSF	PEA	LBD continuum vs HC: elevated across clinical, prodromal and preclinical LBD; predictive of progression
Pereira et al.	2023	CSF	PEA	LBD/AP vs HC (replication): high diagnostic performance (AUCs up to 0.95)
Pereira et al.	2023	Plasma	PEA	LBD/AP vs HC (replication): discriminated LBD and AP from controls (AUCs up to 0.92)
Appleton et al.	2024	CSF	PEA	Prodromal/de novo PD vs HC: increased in prodromal and treatment-naïve PD
Appleton et al.	2024	Plasma	PEA	Prodromal/de novo PD vs HC: no significant plasma changes
Rutledge et al.	2024	CSF	PEA, SomaScan, LC-MS/MS	Early PD vs HC: consistently increased across cohorts and platforms
Rutledge et al.	2024	Urine	PEA, SomaScan, LC-MS/MS	Early PD vs HC: increased in prodromal, de novo and genetic PD
Rutledge et al.	2024	Plasma	PEA, SomaScan, LC-MS/MS	Early PD vs HC: less consistent than CSF and urine
Khosousi et al.	2024	CSF	PEA	PD vs HC: higher DDC associated with lower DaT-SPECT binding
Bolsewig et al.	2025	Plasma	PEA	LBD vs HC: strongly influenced by dopaminergic treatment
Aviolat et al.	2026	CSF	Quant. immunoassay	PD vs HC: increased DDC confirmed by quantitative measurement
Bolsewig et al.	2026	CSF	Quant. immunoassays	PD/DLB vs HC and AD: validated as robust biomarker of synucleinopathies (AUCs up to 0.97)
Bolsewig et al.	2026	CSF	Quant. immunoassays	PD/DLB and AD by α-syn-SAA: associated with clinical synucleinopathies and Lewy body pathology, not isolated AD α-syn-SAA positivity
Bolsewig et al.	2026	Brain	IHC	Neuropathological LBD: colocalized with α-syn within Lewy bodies

a Abbreviations: AD, Alzheimer’s disease; AP, atypical parkinsonian disorders; AUC, area under the curve; CSF, cerebrospinal fluid; DLB, dementia with Lewy bodies; HC, healthy controls; LBD, Lewy body disease; LC-MS/MS, liquid chromatography–tandem mass spectrometry; PD, Parkinson’s disease; PEA, proximity extension assay; SAA, seed amplification assay; α-syn, α-synuclein.

The first quantitative studies showed that the level in CSF is about 100-fold lower than that in plasma, supporting the evidence of a substantial peripheral expression of AADC^10^ (Figure 5). On the other hand, AADC in CSF is probably mostly derived from CNS structures and is either secreted in the CSF or leaked in it due to degeneration of (dopaminergic) neurons. The first study showing differential levels of AADC in biological fluids of LBDs used proximity extension assay (PEA) to measure about 600 proteins in CSF of DLB, Alzheimer’s disease (AD) patients and cognitively unimpaired controls (*n* = 534). AADC showed increased levels in the CSF of DLB patients with respect to AD and control subjects and was the strongest predictor of DLB in a 7-biomarker panel which was able to differentiate DLB from AD with an area under the curve of 0.93.^68^ Paslawski and colleagues reported that CSF AADC, together with midkine, was increased in PD and could support PD diagnosis.^12^ Additionally, CSF AADC levels strongly correlated with dopaminergic treatment and were increased in atypical parkinsonisms.^12^ Pereira et al. then showed in a cohort composed of 682 individuals from the prospective Swedish BioFINDER study, that AADC was increased not only in clinically manifest PD and DLB, but also in individuals with positive α-syn seed amplification assay (SAA) before the appearance of clinical symptoms, suggesting a possible role in predicting progression to clinical LBD over a 3-year period.^11^ In the same study, the CSF levels of AADC were also found to be increased in atypical parkinsonisms.^11^ Subsequently, a large multifluid proteomic study by Rutledge et al. confirmed AADC as a consistent candidate biomarker in early PD, using PEA, SomaScan and LC-MS/MS across CSF, plasma and urine; in this study AADC was increased in CSF and urine from prodromal PD, de novo PD, and genetic risk groups, and CSF AADC correlated with motor severity scores.^69^ All of these studies used semiquantitative techniques to measure AADC in biofluids. More recently, quantitative immunoassays on different platforms were developed to precisely measure the absolute quantity of AADC.^10,70^ Bolsewig et al. developed two quantitative assays for AADC and validated them in five clinical and pathological cohorts. The results showed a good technical performance of the assays, with a good stability and resilience of the protein to preanalytical factors. Furthermore, the study confirmed higher levels of AADC in CSF of DLB and PD patients, a diagnostic performance against controls and AD comparable to previous studies, and an association with α-syn pathology in autopsy-confirmed DLB.^10^ Interestingly, immunohistochemistry of LBD brains showed localization of AADC within Lewy bodies (LBs), possibly confirming a functional association between AADC and α-syn, the main proteinaceous component of LBs.^10^ Altogether, these studies clearly showed the strong biomarker potential of AADC for the diagnosis of LBDs, although several issues remain to be clarified. For instance, the role of AADC in blood remains unclear, despite blood being a biofluid much more accessible than CSF and amenable to longitudinal sampling to track progression or response to treatment. Indeed, Appleton and colleagues found increased levels of AADC in CSF, but not in plasma, in prodromal and de novo PD, while another recent study showed that plasma AADC levels across LBDs were not significantly different before treatment and were strongly influenced by the dosage of dopaminergic treatment as measured by levodopa equivalent daily dose (LEDD).^56,71^ The effect of treatment on peripheral AADC is not limited to protein levels but can also alter the enzyme activity. In a cohort composed of 301 patients with PD or parkinsonisms, treatment with levodopa showed elevation of AADC activity in about 60% of the patients^46^ despite cotreatment with peripheral decarboxylase inhibitors (PDIs). The increase of AADC activity in plasma might be related to different factors including induced peripheral expression due to levodopa or PDIs, activation by phosphorylation or other unknown factors.^72^ Since recent evidence showed that also the protein level is increased upon dopaminergic treatment,^10^ peripheral expression induction might represent the key factor involved in the changes of AADC activity in plasma of treated PD patients. Another issue is related to the biological meaning of the increased AADC levels in CSF of LBDs. Being mainly expressed in the midbrain, AADC may represent a marker of nigrostriatal degeneration but the evidence on the association between CSF AADC levels and dopaminergic deficit is still scarce. A first study showed that higher CSF AADC was associated with lower dopamine transporter binding on DaT-SPECT in the BioPark and PPMI cohorts (measured with PEA), suggesting that CSF AADC may reflect specific nigrostriatal dopaminergic dysfunction rather than only a generic neurodegenerative process.^73^ However, this association was heavily influenced by dopaminergic treatment, making the biomarker change difficult to disentangle from the changes caused by the levodopa administration. In the study by Bolsewig and colleagues the analysis of a subcohort of patients with DaT-SPECT imaging did not evidence a marked association between AADC CSF levels and dopaminergic dysfunction. Similarly, Aviolat et al. developed a quantitative immunoassay for CSF AADC and confirmed its elevation in PD, but did not observe a clear relationship with imaging measures of nigrostriatal degeneration. Interestingly, higher baseline CSF AADC levels were associated with a faster increase in MDS-UPDRS scores during longitudinal follow-up, suggesting that AADC may capture disease progression even if its relationship with the degree of dopaminergic denervation remains not fully understood.^70^ Nevertheless, larger and carefully controlled studies will be required to clearly understand the possible association of AADC levels and dopaminergic dysfunction.

**Figure 5:**
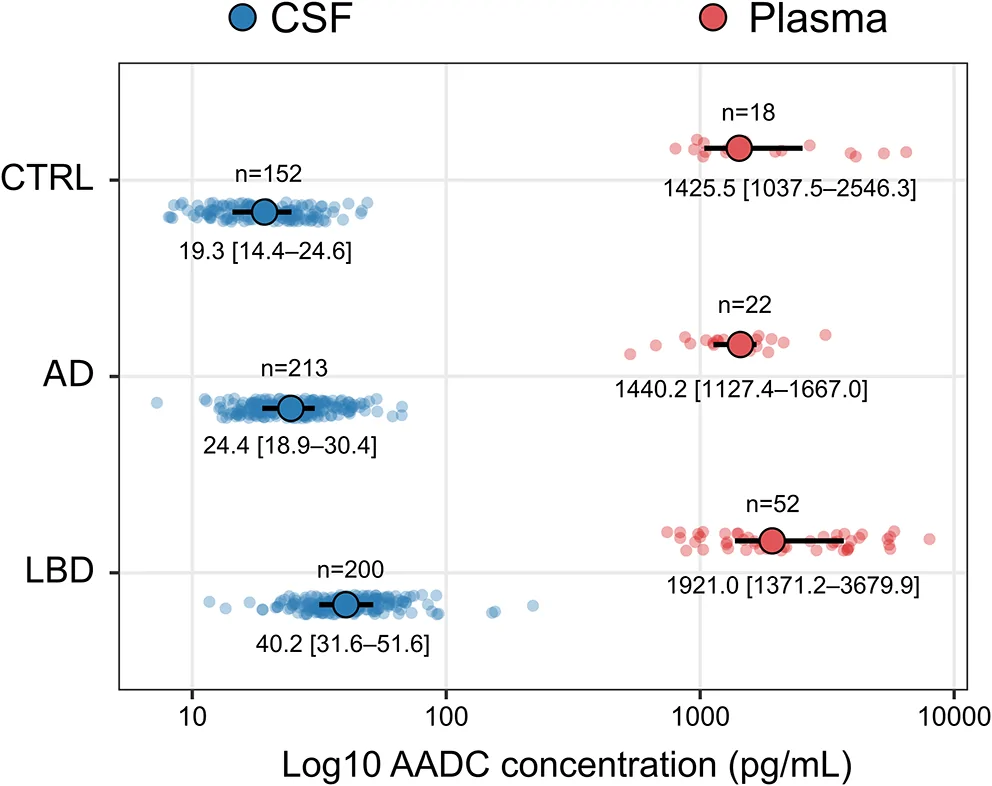
AADC concentrations in cerebrospinal fluid and plasma across diagnostic groups. Distribution of AADC concentrations (log_10_, pg/mL) measured by quantitative immunoassay in cerebrospinal fluid (CSF, blue) and plasma (red) of cognitively unimpaired controls (CTRL), Alzheimer’s disease (AD), and Lewy body disorders (LBD). Large circles and bars indicate group medians with interquartile ranges; the number of samples per group is reported above each distribution. Plasma AADC concentrations are approximately 2 orders of magnitude higher than those in CSF, consistent with a substantial peripheral contribution to the circulating pool. In CSF, AADC is increased in LBD relative to controls and AD, whereas in plasma the elevation in LBD overlaps substantially with the other groups, in line with the strong influence of dopaminergic treatment on peripheral AADC levels.^10,56^ Data from the Source Data file of ref 10

An additional aspect that deserves consideration is the biological relationship between AADC and α-syn pathology. Although increased CSF AADC levels have been consistently reported across the spectrum of LBDs, including prodromal and clinically manifest disease,^11,71^ the available evidence suggests that AADC is unlikely to represent a direct or exclusive marker of α-syn pathology. Indeed, elevated CSF AADC levels have also been observed in atypical parkinsonian syndromes,^11,12^ and recent studies showed that AADC does not clearly distinguish AD patients with positive versus negative α-syn seed amplification assays.^10^ Therefore, while the recent demonstration of AADC colocalization within Lewy bodies supports a biological connection between AADC and α-syn aggregation,^10^ these findings indicate that AADC may reflect broader processes related to dopaminergic dysfunction, neuronal stress, or compensatory responses rather than the burden of α-syn pathology itself. CSF AADC therefore stands as a promising biomarker for LBDs, with potential applications in differential diagnosis and in the assessment of dopaminergic involvement. Plasma AADC, by contrast, appears to be strongly influenced by peripheral expression changes induced by dopaminergic treatment and currently requires further investigation to clarify its clinical utility. Future studies should also determine to what extent CSF AADC reflects disease stage, nigrostriatal degeneration, and the burden of α-syn pathology, as well as how these relationships are modified by treatment. Notably, the recent demonstration of AADC colocalization within Lewy bodies, together with the central role of both AADC and α-syn in PD pathophysiology, raises the possibility of biologically meaningful interactions between these two proteins. Whether AADC and α-syn act synergistically in disease mechanisms, influence each other’s aggregation, trafficking, or cellular toxicity, or simply converge within the same pathological structures remains unknown. Addressing these questions may not only improve the biological interpretation of AADC as a biomarker, but also provide new insights into the molecular mechanisms underlying LBDs. Finally, due to the role of AADC in serotonin synthesis, its diagnostic performance should be evaluated also in neuropsychiatric disorders characterized by serotonergic dysfunction, such as major depressive disorder, schizophrenia, and related conditions. This is very relevant, since we do not currently have robust biological indicators that can support their diagnosis or treatment monitoring.

## Conclusions

Over the last 15 years, human DOPA decarboxylase has moved from the periphery to the center stage of attention in metabolic and neurodegenerative disorders. The structural biology of the human enzyme, from the apo-open conformation described by Giardina, to the asymmetric holo-dimer characterized by Bisello, to the high-resolution carbidopa-bound complex resolved by Baine, provides a framework on which to anchor the regulatory and pharmacological observations accumulated over decades. In parallel, the biomarker literature has positioned AADC as a promising candidate biomarker for LBDs in human biofluids. Several molecular details remain to be elucidated to fully understand the impact of AADC in health and disease. For instance, the pharmacology of carbidopa remains mechanistically ambiguous, the relationship between α-syn and AADC has not been studied systematically and a global view on the interactions between human and microbiota AADCs has not been evaluated as a single system. Taken together, these observations characterize AADC not only as a key player in monoamine biosynthesis, but as a dynamic enzyme whose function is shaped by conformation, post-translational modifications and protein–protein interactions. Defining how all of these layers fit into a single picture will be essential to understand if AADC can be translated from an emerging candidate biomarker to a clinically validated indicator of synucleinopathies.
